# The Association between Toxoplasma gondii Infection and Risk of Parkinson's Disease: A Systematic Review and Meta-Analysis

**DOI:** 10.1155/2019/8186017

**Published:** 2019-02-25

**Authors:** Zonglei Zhou, Ruzhen Zhou, Kunpeng Li, Wen Wei, Zengqiao Zhang, Yan Zhu, Rongsheng Luan

**Affiliations:** ^1^Department of Epidemiology and Biostatistics, Sichuan University West China School of Public Health, Chengdu 610041, Sichuan Province, China; ^2^Department of Anorectal Surgery, Changhai Hospital of Shanghai, Shanghai 200433, China; ^3^Department of Neurorehabilitation, Shanghai Second Rehabilitation Hospital, Shanghai 200441, China; ^4^School of Rehabilitation Science, Shanghai University of Traditional Chinese Medicine, Shanghai 201203, China

## Abstract

**Background:**

Several studies have investigated the association between Toxoplasma gondii (T. gondii) infection and risk of Parkinson's disease (PD) with inconsistent results. Clarifying this relation might be useful for better understanding of the risk factors and the relevant mechanisms of PD, thus a meta-analysis was conducted to explore whether exposure to T. gondii is associated with an increased risk of PD.

**Methods:**

We conducted this meta-analysis according to the Preferred Reporting Items for Systematic Reviews and Meta-Analyses (PRISMA) statement. A rigorous literature selection was performed by using the databases of PubMed, Embase, Web of Science, Cochrane Library, and ScienceDirect. Odds ratio (OR) and corresponding 95% confidential interval (CI) were pooled by using fixed-effects models. Sensitivity analysis, publication bias test, and methodological quality assessment of studies were also performed.

**Results:**

Seven studies involving 1086 subjects were included in this meta-analysis. Pooled data by using fixed-effects models suggested both latent infection (OR, 1.17; 95% CI, 0.86 to 1.58;* P*=0.314) and acute infection (OR, 1.13; 95% CI, 0.30 to 4.35;* P*=0.855) were not associated with PD risk. Stable and robust estimates were confirmed by sensitivity analysis. No publication bias was found by visual inspection of the funnel plot, Begg's, and Egger's test.

**Conclusions:**

This meta-analysis does not support any possible association between T. gondii infection and risk of PD. Researches are still warranted to further explore the underlying mechanisms of T. gondii in the pathogenesis of PD and their causal relationship.

## 1. Introduction

With an estimated prevalence of 315 per 100,000 worldwide, Parkinson's disease (PD) is the second most common neurodegenerative disorder next to Alzheimer's disease [1, 2]. For the deficiency of dopamine, individuals with PD are inclined to suffer from motor dysfunction and nonmotor disturbance [3, 4]. Patients with PD tend to have a short time of survival [5] and low quality of life [6], and the risk of mortality increases with disease duration [7]. Neurodegenerative disorders like PD are projected to surpass cancer as the leading cause of death by 2040 [8]; thus great emphasis should be placed on the prevention of PD in our aging society. However, the etiology of PD remains unclear.

Toxoplasma gondii (T. gondii) is an intracellular protozoan parasite that causes a zoonotic disease known as toxoplasmosis. Over thirty percent of the world population was infected with T. gondii, and it is viewed as the most prevalent infection in humans [9]. Primarily carried by cats and other felines, T. gondii can infect most mammals. Humans might be infected with T. gondii by consumption of meat containing tissue cysts, intake of oocysts, and maternal-neonatal transmission [10, 11]. In addition, T. gondii could be transmitted by blood transfusion, solid organ, or hematopoietic stem cell transplantation [12]. Infected population usually remains asymptomatic for the effective immune responses, and T. gondii tissue cysts are predominantly formed in the brain and muscles in latent toxoplasmosis [13]. However, when the immune responses of hosts weaken, tissue cysts rupture followed by the release of bradyzoites [14]. These recrudescent infections make ways for rapidly dividing tachyzoite stagel thus toxoplasmic encephalitis and neurological damage might be incurred [15, 16]. Decreased psychomotor performance was observed in both infected humans [17] and animals [18]. Meantime, the onset and severity of PD were reported to be associated with body inflammatory responses [19, 20], and anti-inflammatory therapies exhibited favorable effects on protection of dopaminergic neurons [21]. However, T. gondii can also increase the production of dopamine [22]. Animal experiments indicated that mice infected with T. gondii had higher dopamine concentration in the brain compared with uninfected mice [23, 24]. These effects might be associated with tyrosine hydroxylase encoded in T. gondii genome, which gets involved in the biosynthesis of dopamine [25].

So far, inconsistent conclusions of several epidemiological studies investigating the association between T. gondii infection and PD risk have been reported. Ramezani et al. [26] suggested T. gondii infection contributed to an increased risk of PD, and Miman et al. [27] also considered T. gondii might be involved in the development of PD. To the contrary, Alvarado-Esquivel et al. [28] and Oskouei et al. [29] indicated no association was observed between T. gondii infection and PD, Fallahi et al. [11] found that T. gondii infection could not be a risk factor for PD. To date, and no meta-analysis was conducted to combine the available evidence. Giving the controversial pathogenetic mechanisms and population-based studies, we performed a meta-analysis to shed light on this issue according to the Preferred Reporting Items for Systematic Reviews and Meta-Analyses (PRISMA) guidelines [30].

## 2. Materials and Methods

### 2.1. Literature Search

A systematic literature search was conducted in five English electronic databases from their inceptions to October 2018. The databases included PubMed, Embase, Web of Science, Cochrane Library, and ScienceDirect. Keywords for literature search were listed as follows: “Toxoplasma gondii”, “toxoplasmosis”, “Parkinson's disease”, and “Parkinsonism”. Synonyms and variations of keywords were applied to ensure a comprehensive search. Reference lists of relevant reviews were also screened for potentially eligible literature. The detail search strategy for PubMed database was available in Table 1.

### 2.2. Publication Selection

Two reviewers independently screened the titles and abstracts of retrieved articles. For publications whose eligibility could not be determined by the above process, full-text reading was performed for further evaluation. Studies meeting the following criteria were considered eligible: (a) case-controlled or cohort studies investigating the relationship between T. gondii infection and risk of PD; (b) the exposure was T. gondii infection diagnosed by positive serum anti-T. gondii Ig G antibodies or IgM antibodies, which respectively represented latent and acute infection [31]; (c) the interesting outcome was PD; (d) relative risk (RR), hazard ratio (HR) or odds ratio (OR) with 95% confidential interval (CI) were reported or could be calculated.

Studies were excluded if they were (a) reference papers, case reports, reviews, letters, and methodological or nonpopulation-based researches; (b) studies discussing nontargeted outcomes or exposures; (c) studies with incomplete data even if attempts were made to contact authors.

Any disputes were settled by discussion or referral to a third reviewer.

### 2.3. Data Extraction and Methodological Quality Assessment

The related data of included studies were independently extracted by two investigators, with a standardized data extraction checklist. Extracted data of individual study included first author, publication year, region, study design, number of cases and controls, demographic features of participants (disease status, age and male%), measurement of exposure, HR, RR, or OR with corresponding 95% CI, adjusted factors.

In this review, Newcastle-Ottawa quality assessment scale (NOS) [32] was applied to appraise the methodological quality of studies. NOS was used to evaluate the methodological quality of non-randomized studies, including cohort and case-controlled studies. With a maximum of 9 points, studies with a NOS score of ≥6 points were considered to be of high quality [33].

Disagreements regarding data extraction and methodological quality appraisal were resolved by a third investigator.

### 2.4. Data Synthesis and Statistical Analysis

The association between T. gondii infection and risk of PD was measured by OR. The HR and RR were considered to be equivalent to OR for the low incidence of PD in the population [34]. Heterogeneity across studies was tested by Cochran* Q* statistic, with a significant level of 0.1. We also evaluated statistical heterogeneity by using *I*^2^ statistic, whose cut-off values of 25%, 50%, and 75% were, respectively, viewed as low, medium, and high heterogeneity [35]. We synthesized OR and 95% CI of each study by using a fixed-effects model if no or low heterogeneity existed, and data were combined with a random-effects model if there was medium heterogeneity. Otherwise, narrative review was performed. Publication bias was assessed by visual observation of funnel plot and Egger's publication bias plot; we also used Begg's rank correlation test and Egger's linear regression test to make a quantitative judgment if appropriate. If publication bias existed in studies, “trim and fill” method was used to further evaluate the effects of publication bias in this review [36]. Sensitivity analysis was performed to explore the effects of methodological quality of studies on the stability of pooled results. We used Stata 12.0 software to pool data and create relevant plots.

## 3. Results

### 3.1. Literature Search Results

**Figure 1:**
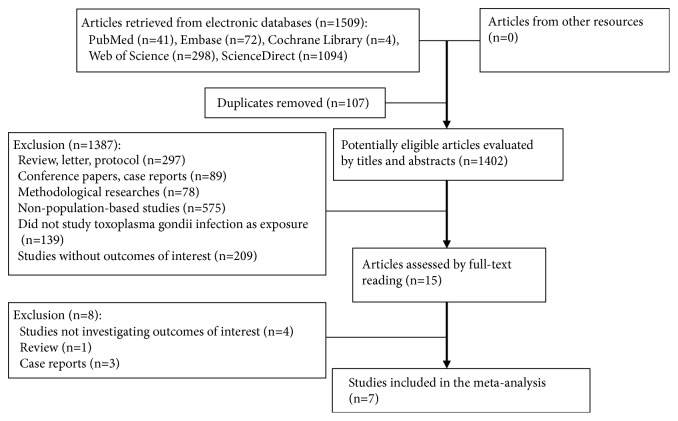
Flow diagram of literature selection.

Literature screening process and corresponding exclusion reasons were illustrated in Figure 1. We retrieved 1509 articles from PubMed, Embase, Web of Science, Cochrane Library, and ScienceDirect, including 107 duplicates. The majority of retrieved articles were excluded by scrutinizing titles and abstracts; only 15 potentially eligible literatures remained for further evaluation. Of 15 studies, four were excluded for irrelevant outcomes and four were not cohort or case-controlled studies (one review, three case reports). Eventually, seven studies [11, 26–29, 37, 38] (eight comparison groups) involving 1086 participants were included in this meta-analysis.

### 3.2. Characteristics and Methodological Quality of Included Studies

Of the seven studies, three originated from Iran [11, 26, 29], three from Turkey [27, 37, 38], and one from Mexico [28]. Published between 2010 and 2017, all studies were based on case-controlled design. Sample size of included studies ranged from 95 to 260 and mean age ranged from 62 to 76.3 years. Exposure of cases and controls measured by anti-T. gondii Ig G antibodies was available in all studies, while only three studies [11, 26, 28] reported exposure of cases and controls measured by anti-T. gondii Ig M antibodies. All studies were deemed to be of high quality, except one study [38]. Characteristics and methodological quality of individual study were presented in Table 2.

### 3.3. T. gondii Infection and Risk of PD

**Figure 2:**
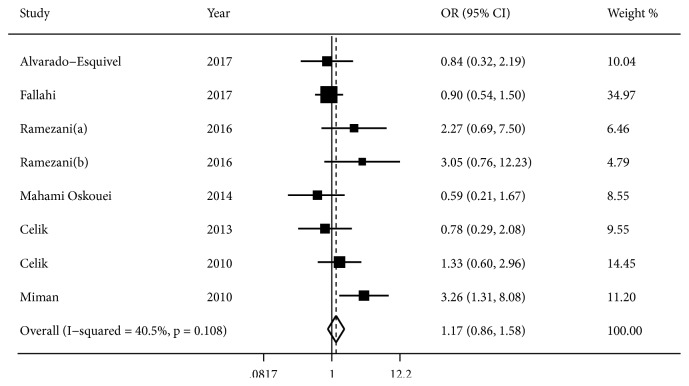
Forest plot of association between latent infection of T. gondii and risk of PD.

Seven studies [11, 26–29, 37, 38] (eight comparison groups) investigated the association between latent infection of T. gondii and risk of PD (Figure 2). With low to medium heterogeneity across studies (*I*^2^=40.5%,* P*=0.108), results of pooled fixed-effects analysis indicated latent infection of T. gondii was not associated with risk of PD (OR, 1.17; 95% CI, 0.86 to 1.58;* P*=0.314). After excluding low-quality studies, sensitivity analysis demonstrated the similar results (OR, 1.14; 95% CI, 0.82 to 1.59;* P*=0.422), suggesting pooled results were stable and robust.

**Figure 3:**
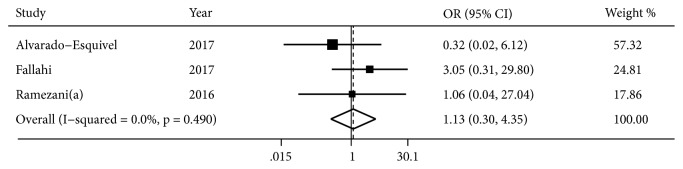
Forest plot of association between acute infection of T. gondii and risk of PD.

Three studies [11, 26, 28] reported the effect of acute infection of T. gondii on PD risk (Figure 3). There was no heterogeneity across studies (*I*^2^=0.0%,* P*=0.490); a fixed-effects model was used to synthesize data. Pooled results suggested no association was observed between acute infection of T. gondii and PD risk (OR, 1.13; 95% CI, 0.30 to 4.35;* P*=0.855).

### 3.4. Publication Bias

**Figure 4:**
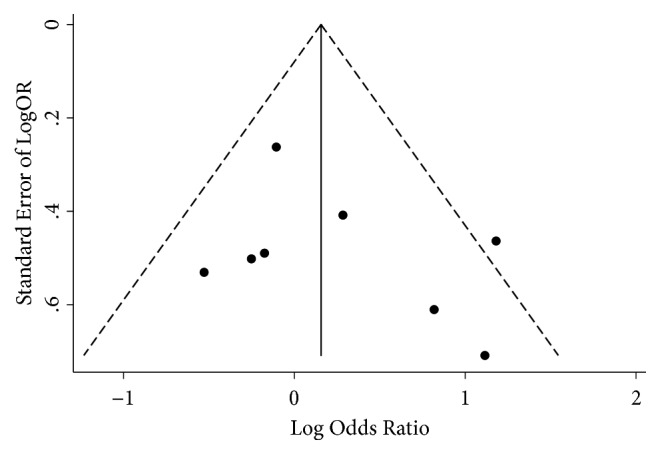
Funnel plot with pseudo 95% CI.

**Figure 5:**
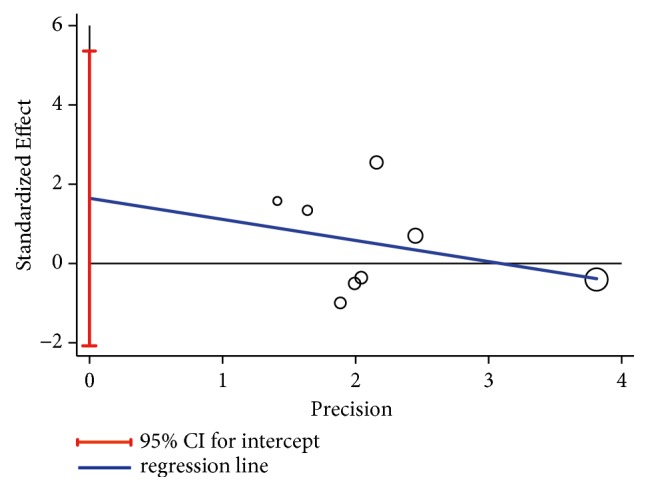
Egger's publication bias plot. The intercept of Egger's regression represented publication bias.

Some asymmetry was observed from funnel plot (Figure 4); however, both Begg's rank correlation test (*Z*=0.37,* P*=0.711) and Egger's linear regression test (*t*=1.09,* P*=0.316, Figure 5) showed that no publication bias existed in included studies. And there was no change in the results of pooled fixed-effects analysis corrected by using “trim and fill” method.

## 4. Discussion

Characterized by motor dysfunction and nonmotor symptoms, PD is generally considered to be a multifactorial disease intrigued by an interaction between genetic and environmental factors [11]. From a pathophysiological perspective, progressive deterioration of dopaminergic neurons, especially in the substantia nigra, gets involved in pathogenetic changes of PD [4]. Since the central nervous system is the most vulnerable site in individuals infected with T. gondii [39], long-standing interest has been aroused in exploring the association between exposure to T. gondii and central nervous system diseases, including cryptogenic epilepsy [40], migraine [41], Alzheimer's disease [42], and schizophrenia [43]. Although the association between exposure to T. gondii and PD risk was reported in several studies [11, 26–29, 37, 38], the conclusions of these studies were conflicting.

Our meta-analysis systematically reviewed all existing studies investigating the association between T. gondii infection and risk of PD, and seven case-controlled studies involving 1086 subjects were included in the pooled analysis. We found neither T. gondii latent infection (OR, 1.17; 95% CI, 0.86 to 1.58;* P*=0.314) nor acute infection (OR, 1.13; 95% CI, 0.30 to 4.35;* P*=0.855) was associated with risk of PD.

To the best of our knowledge, this is the first meta-analysis investigating the association between T. gondii infection and PD risk. Eligible studies were selected by applying comprehensive and rigorous inclusion criteria. For each study, NOS was applied to evaluate the quality of method, and we found all identified studies were deemed to be of high quality, except for one study [38]. To ensure stable results, we also performed sensitivity analysis by combining data after excluding low-quality studies, and no obvious difference was found. Although the funnel plot showed some asymmetry, publication bias test was not significant. Also, “trim and fill” method was used to provide a comprehensive appraisal of the potential effects of publication bias. Both sensitivity analysis and statistical test of publication bias indicated our results were robust and reliable.

T. gondii can cause excessive expression of cytokines and chemokines as well as activation of astrocytes; these reactions might facilitate proinflammatory responses [44]. Proinflammatory cytokines could be neuroprotective, but proinflammatory cytokines with long-term or sustained increase might exert adverse effects on dopaminergic neurons [45]. Previous studies [46, 47] reported a decreased tendency of inflammatory responses was found in latent stage of T. gondii infection, and degeneration of neurons did not commonly occur during chronic infection. Despite dopaminergic neurons degeneration and proinflammatory responses resulting from T. gondii, T. gondii can also produce tyrosine hydroxylase encoded in two genes of its genome [25]. Generated by T. gondii during the formation of the bradyzoites stage, this enzyme can facilitate the rate-limiting step of dopamine biosynthesis [48], whose deficiency was considered to be related with PD. Based on the above pathophysiological mechanisms, it was hypothesized that dopamine produced by T. gondii might make some compensation for dopaminergic neurons degeneration caused by T. gondii infection; thus the effects of T. gondii infection on the onset of PD might be weakened for the offset of these two forms of influence.

Several limits should be considered in the interpretation of this review. First, our conclusions might be influenced since subjects in different regions usually had different genetic factors, environmental exposures, and lifestyles. Second, all studies in this review were based on case-controlled design; thus only association between exposure to T. gondii and PD risk could be investigated, not causal relationship. Third, potential language bias might exist in our review, as eligible studies were restricted to literature in English language. Fourth, subgroup analysis was not conducted due to limited information.

## 5. Conclusions

This review does not suggest any association between T. gondii infection and development of PD. The pathogenic mechanisms of T. gondii in PD still remain incompletely clear; further researches are required to figure out the underlying mechanisms. Moreover, well-conducted and large cohort studies are warranted to further investigate the causal relationship between exposure to T. gondii and the development of PD. Whether treatment of T. gondii infection could effectively prevent or delay the progress of PD is also suggested to be tested in long-term and high-quality intervention studies.

## Figures and Tables

**Table 1:** The search strategy for PubMed database.

Search	Query	No. of retrieved articles

#1	Toxoplasmas [Title/Abstract] OR Toxoplasma gondii [Title/Abstract] OR Toxoplasma gondius [Title/Abstract] OR toxoplasmosis [Title/Abstract] OR toxoplasmoses [Title/Abstract]	23829
#2	Toxoplasmas [Mesh]	12692
#3	#1 OR #2	25291
#4	Parkinson disease [Title/Abstract] OR idiopathic Parkinson disease [Title/Abstract] OR Parkinsonism [Title/Abstract] OR primary Parkinsonism [Title/Abstract] OR Parkinsonian disorder [Title/Abstract] OR Parkinsonian syndrome [Title/Abstract] OR Parkinson [Title/Abstract] OR PD [Title/Abstract] OR paralysis agitans [Title/Abstract] OR lewy body [Title/Abstract]	138592
#5	Parkinson disease [Mesh]	59868
#6	#4 OR #5	163560
#7	#3 AND #6	41

**Table 2:** Characteristics and methodological quality of included studies for analysis of T. gondii infection and risk of PD.

Studies; years of publication; country	Study design	Participants		Number; age (years); male%		Exposure measures	OR (95% CI)	Adjustments	NOS score
		Case	Control	PD	Control				

Alvarado-Esquivel et al [28]; 2017; Mexico	Case-control	Patients with PD	Subjects without PD	65; 69.08±11.39; 46.15	195; 68.56±10.08; 46.15	E_*a*_: latent infection, positive serum anti-T. gondii IgG antibodies; E_*b*_: acute infection, positive serum anti-T. gondii IgM antibodies; Non-exposure: no infection, negative serum anti-T. gondii IgG or IgM antibodies	E_*a*_: 0.84 (0.32, 2.18)^*∗*^ E_*b*_: 0.33 (0.02, 6.12)^*∗∗*^	Age, gender	8
Fallahi et al [11]; 2017; Iran	Case-control	PD patients	Healthy individuals	115; 75.2±13.1; NA	115; 74.1±14.4; NA		E_*a*_: 0.90 (0.54, 1.51)^*∗*^ E_*b*_: 3.02 (0.31, 29.80)^*∗*^	Age, sex and place of residence	7
Ramezani (a) et al [26]; 2016; Iran	Case-control	Individuals with IPD	Healthy subjects;	41; 76.3±6; 78	69; 62±8; 78.2;		E_*a*_: 2.27 (0.69, 7.5)^*∗∗*^ E_*b*_: 1.06 (0.04, 27.04)^*∗∗*^	Sex, age and socioeconomic status	8
Ramezani (b) et al [26]; 2016; Iran			Patients without PD		40; 64.6±3.5; 77.5;	E_*a*_: latent infection, positive serum anti-T. gondii IgG antibodies; Non-exposure: no infection, negative serum anti-T. gondii IgG or IgM antibodies	E_*a*_: 3.05 (0.76, 12.24)^*∗∗*^		
Oskouei et al [29]; 2014; Iran	Case-control	Parkinson's patients	Healthy subjects	75; 63.7±11.3; 77.3	75; 63.4±11.6; NA		E_*a*_: 0.59 (0.20, 1.60)^*∗*^	Age, gender, residency, education, cat keeping, using raw or undercooked meat and egg	7
Celik et al [37]; 2013; Turkey	Case-control	Patients with IPD	Healthy individuals	50; 65.6±10.2; 64	50; 65.6±30.4; 58	E_*a*_: latent infection, positive serum anti-T. gondii IgG antibodies; Non-exposure: no infection, negative serum anti-T. gondii IgG or IgM antibodies	E_*a*_: 0.78 (0.29, 2.08)^*∗∗*^	Age, gender	7
Celik et al [38]; 2010; Turkey	Case-control	Patients with IPD	Healthy individuals	50; 63.39±13.21; 60	45; 62.38±11.55; 64		E_*a*_: 1.33 (0.60, 2.96)^*∗*^	Age	5
Miman et al [27]; 2010; Turkey	Case-control	Patients with PD	Healthy individuals	52; 66.01±12.14; 61.54	40; 62.42±5.93; 52.5		E_*a*_: 3.26 (1.31, 8.09)^*∗∗*^	Age, gender, socioeconomic status	6

T. gondii: Toxoplasma gondii; PD: Parkinson's disease; NOS: Newcastle-Ottawa quality assessment scale; NA: not available; IPD: idiopathic Parkinson's disease; IgG: immunoglobulin G; IgM: immunoglobulin M.

∗Adjusted value obtained from original reports; ∗∗value calculated according to counts of event and total number of two groups in individual study.

Data were presented as mean ± standard deviation where appropriate.
